# Supplementary material for: Structural and Functional Characterization of the Vacuolar-Type Na+, K+/H+ Antiporter NHX1 from Rice (Oryza sativa L.)
Source: Biomolecules. 2025 Oct 27;15(11):1513. doi: 10.3390/biom15111513 (PMC12649843; doi:10.3390/biom15111513)
Supplement: Supplementary file 1 [file biomolecules-15-01513-s001.zip › Supplementary Materials (Figure S1, Table S1, Table S2, Table S3).pdf]

Supplementary Materials

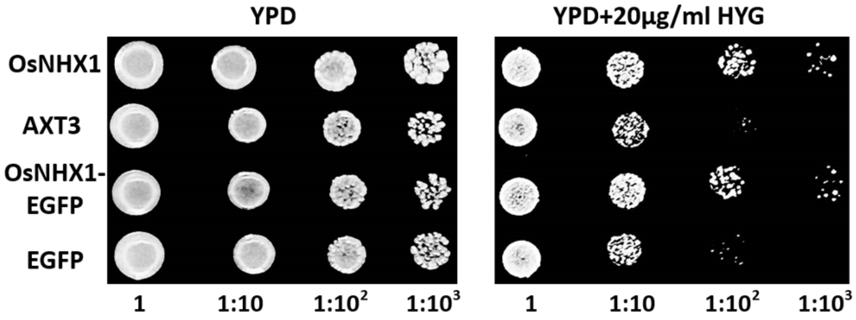

Figure S1. Growth of AXT3 strains expressing OsNHX1 and OsNHX1-EGFP was evaluated on YPD medium, with and without supplementation of hygromycin B. The OsNHX1 open-reading frames were cloned into the plasmid pYPGE15 and the plasmid pYPGE15-EGFP without the stop codon. These constructs were introduced into the yeast mutant strain AXT3. The empty pYPGE15 and pYPGE15-EGFP were used as negative controls, respectively. 4 μL of 10-fold serial dilutions of these strains from saturated cultures were spotted onto the YPD plates with or without hygromycin B (20μg/mL).

Table S1. Primers used in this work.

Primer	Sequence	Use
OsNHX1-F	ATGGGGATGGAGGTGGCGGCGG	Cloning of OsNHX1 (without the stop codon were fused with the EGFP)
OsNHX1-R	CTATCTTCCTCCATGGCTCTGCTCG	
E43Q-F	CTCGGCCACCTCCTCCAGGAGAATCGCTGG	Point mutations of the conserved negatively charged residues of OsNHX1
E43Q-R	CTGGAGGAGGTGGCCGAGGACGATGCAGGC	
E50Q-F	AATCGCTGGGTCAATCAGTCCATCACCGCG	
E50Q-R	CTGATTGACCCAGCGATTCTCCTCGAGGAG	
E81Q-F	TTATTCGTCTTCAGTCAGGATCTCTTCTTC	
E81Q-R	CTGACTGAAGACGAATAAGTGCGAGCTCTT	
D147N-F	ACGCTGGATGTAGGAAATTTTCTTGCAATT	
D147N-R	ATTTCCTACATCCAGCGTTCCAATGTTCAT	
D159N-F	ATCTTTTCTGCGACAAATTCTGTCTGCACA	
D159N-R	ATTTGTCGCAGAAAAGATGGCTCCAATTGC	
D170N-F	CAGGTCCTCAATCAGAATGAGACACCCTTT	
D170N-R	ATTCTGATTGAGGACCTGCAATGTGCAGAC	
E171Q-F	GTCCTCAATCAGGATCAGACACCCTTTTTG	
E171Q-R	CTGATCCTGATTGAGGACCTGCAATGTGCA	
E182Q-F	AGTCTGGTATTCGGTCAAGGTGTTGTGAAC	
E182Q-R	TTGACCGAATACCAGACTGTACAAAAAGGG	
D187N-F	GAAGGTGTTGTGAACAATGCTACATCAATT	
D187N-R	ATTGTTCACAACACCTTCACCGAATACCAG	
D250N-F	GGAAGGCATTCTACTAACCGTGAGGTTGCC	

D250N-R	GTTAGTAGAATGCCTTCCAATGTATAGCTT	
E252Q-F	CATTCTACTGACCGTCAGGTTGCCCTTATG	
E252Q-R	CTGACGGTCAGTAGAATGCCTTCCAATGTA	
E268Q-F	TCATATATGCTGGCTCAGTTGCTAGATTTG	
E268Q-R	CTGAGCCAGCATATATGAAAGGTAAGCCAT	
E313Q-F	CTGTCCTTCATTGCTCAGACTTTTCTCTTC	
E313Q-R	CTGAGCAATGAAGGACAGAGTTGCAAATGC	
D323N-F	CTGTATGTTGGGATGAATGCATTGGATATT	
D323N-R	ATTCATCCCAACATACAGGAAGAGAAAAGT	
D326N-F	GGGATGGATGCATTGAATATTGAAAAATGG	
D326N-R	ATTCAATGCATCCATCCCAACATACAGGAA	
R46A-F	CTCCTCGAGGAGAATGCCTGGGTCAATGAG	Point mutations of the conserved positively charged residues of OsNHX1
R46A-R	GGCATTCTCCTCGAGGAGGTGGCCGAGGAC	
K101A-F	GCAGGTTTTCAGGTAGCGAAAAAGCAATTC	
K101A-R	CGCTACCTGAAAACCTGCATTGAAGATGAT	
K102A-F	GGTTTTCAGGTAAAGGCAAAGCAATTCTTC	
K102A-R	GCCTTTACCTGAAAACCTGCATTGAAGAT	
K103A-F	TTTTCAGGTAAAGAAAGCGCAATTCTTCCGG	
K103A-R	CGCTTTCTTTACCTGAAAACCTGCATTGAA	
K240A-F	AGTGCATACATAATCGCGAAGCTATACATT	
K240A-R	CGCGATTATGTATGCACTGAGCAATCCAGC	
R246A-F	AAGCTATACATTGGAGCGCATTCTACTGAC	
R246A-R	CGCTCCAATGTATAGCTTCTTGATTATGTA	
R251A-F	AGGCATTCTACTGACGCTGAGGTTGCCCTT	
R251A-R	AGCGTCAGTAGAATGCCTTCCAATGTATAG	
H287A-F	GGTATTGTAATGTCAGCTTACACTTGGCAT	
H287A-R	AGCTGACATTACAATACCACAGAAGAATAC	
R355A-F	TTGGTTCTGATTGGAGCAGCTGCTTTTGTA	
R355A-R	TGCTCCAATCAGAACCAATCCTAGCAAAAT	
R392A-F	TGGGCTGGGCTGATGGCAGGAGCTGTGTCG	
R392A-R	TGCCATCAGCCCAGCCCACCATATTACAAC	
K438A-F	TTTGGGATGATGACAGCGCCATTGATCAGG	
K438A-R	CGCTGTCATCATCCCAAATACCATAGTGCT	
P90A-F	TTCATCTACCTCCTCGCTCCGATCATCTTC	
P90A-R	AGCGAGGAGGTAGATGAAGAAGAGATCCTC	
P91A-F	ATCTACCTCCTCCCTGCGATCATCTTCAAT	
P91A-R	CGCAGGGAGGAGGTAGATGAAGAAGAGATC	
T158A-F	GCCATCTTTTCTGCGGCAGATTCTGTCTGC	
T158A-R	TGCCGCAGAAAAGATGGCTCCAATTGCAAG	

S160A-F	CTTTTCTGCGACAGATGCTGTCTGCACATTG	Point mutations of the conserved structure-related residues of OsNHX1
S160A-R	AGCATCTGTCGCAGAAAAGATGGCTCCAATT	
S160D-F	CTTTTCTGCGACAGATGATGTCTGCACATTG	
S160D-R	ATCATCTGTCGCAGAAAAGATGGCTCCAATT	
S160K-F	CTTTTCTGCGACAGATAAAGTCTGCACATTG	
S160K-R	TTTATCTGTCGCAGAAAAGATGGCTCCAATT	
S160P-F	CTTTTCTGCGACAGATCCTGTCTGCACATTG	
S160P-R	AGGATCTGTCGCAGAAAAGATGGCTCCAATT	
S273A-F	GAGTTGCTAGATTTGGCCGGCATTCTCACC	
S273A-R	GGCCAAATCTAGCAACTCAGCCAGCATATA	
S273D-F	GAGTTGCTAGATTTGGACGGCATTCTCACC	
S273D-R	GTCCAAATCTAGCAACTCAGCCAGCATATA	
S273K-F	GAGTTGCTAGATTTGAAAGGCATTCTCACC	
S273K-R	TTTCAAATCTAGCAACTCAGCCAGCATATA	
S273P-F	GAGTTGCTAGATTTGCCCGGCATTCTCACC	
S273P-R	GGGCAAATCTAGCAACTCAGCCAGCATATA	

Table S2. Highly conserved charged residues in the model of OsNHX1 are detailed in the Table.

OsNHX1	Location	Conservation score	
Glu43	TM1	7	Negatively charged residues
Glu50	TM2	8	
Glu81	TM3	7	
Asp147	TM5	7	
Asp159	TM5	9	
Asp170	TM5/6	6	
Glu171	TM5/6	7	
Glu182	TM6	9	
Asp187	TM6	9	
Asp250	TM8	8	
Glu252	TM8	9	
Glu268	TM8	9	
Glu313	TM10	9	
Asp323	TM10	8	
Asp326	TM10	8	
Arg46	TM1/2	8	
Lys101	TM3/4	8	
Lys102	TM3/4	8	
Lys103	TM3/4	8	

Lys240	TM7	8	Positively charged residues
Arg246	TM7/8	7	
Arg251	TM8	8	
His287	TM9	9	
Arg355	TM11	9	
Arg392	TM12	9	
Lys438	TM13	7	
Pro90	TM3	9	Structure-related residues
Pro91	TM3	9	
Thr158	TM5	9	
Ser160	TM5	9	
Ser273	TM8/9	9	

Table S3. The key amino acid sites responsible for ion transport in CPA1 family members. Summary of key amino acid residues in the crystal structures of OsSOS1 and NHE1, and the model of OsNHX1.

Location	OsNHX1	NHE1 [1] [2]	OsSOS1 [3]
TM2	Glu50	Glu131	
TM3	Pro90<br>Pro91	Pro167<br>Pro168	Pro82
TM3		Asp172	Glu87
TM5	Thr158		Thr146
TM5	Asp159	Asp238	Asp147
TM5	Ser160		
TM6	Glu182	Glu262	Glu171
TM6		Ser263	Ser172
TM6	Asp187	Asp267	Asp176
TM8/9	Ser273	Ser351	Ser258
TM10	Glu313	Glu391	

References

1. Dong, Y.; Gao, Y.; Ilie, A.; Kim, D.; Boucher, A.; Li, B.; Zhang, X. C.; Orlowski, J.; Zhao, Y. Structure and mechanism of the human NHE1-CHP1 complex. *Nat. Commun.* **2021**, 12(1), 3474.
2. Landau, M.; Herz, K.; Padan, E.; Ben-Tal, N. Model structure of the Na^+/H^+ exchanger 1 (NHE1) functional and clinical implications. *J. Biol. Chem.* **2007**, 282, 37854-37863.
3. Zhang, X. Y.; Tang, L. H.; Nie, J. W.; Zhang, C. R.; Han, X.; Li, Q. Y.; Qin, L.; Wang, M. H.; Huang, X. H.; Yu, F. F.; Su, M.; Wang, Y. C.; Xu, R. M.; Guo,Y.; Xie, Q.; Chen, Y. H. Structure and activation mechanism of the rice Salt Overly Sensitive 1 (SOS1) Na+/H+ antiporter. *Nat. Plants* **2023**, 9(11), 1924-1936.
