# Supplementary material for: Structural and Functional Characterization of the Vacuolar-Type Na+, K+/H+ Antiporter NHX1 from Rice (Oryza sativa L.)
Source: Biomolecules. 2025 Oct 27;15(11):1513. doi: 10.3390/biom15111513 (PMC12649843; doi:10.3390/biom15111513)
Supplement: Supplementary file 1 [file biomolecules-15-01513-s001.zip › Supplementary Materials (Original images of Western blot).pdf]

Original image

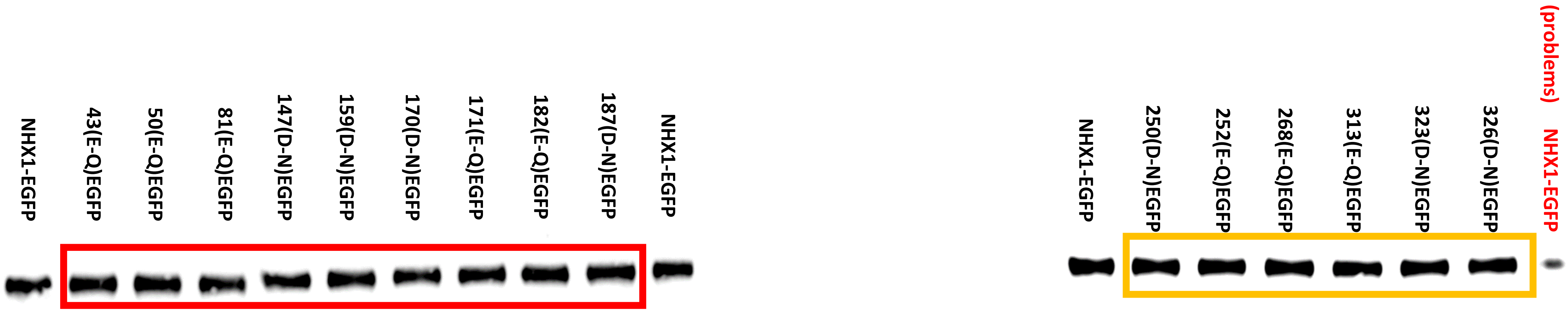

The microsomal membrane proteins (25 µg) were separated by SDS-PAGE, the SDS-PAGE gel (between 75kDa-100kDa) is cut, and was subjected to Western blot, using GFP antibody.

The sample of 43(E-Q)EGFP, 50(E-Q)EGFP, 81(E-Q)EGFP, 147(D-N)EGFP, 159(D-N)EGFP, 170(D-N)EGFP, 171(E-Q)EGFP, 182(E-Q)EGFP, 187(D-N)EGFP, 250(D-N)EGFP, 252(E-Q)EGFP, 268(E-Q)EGFP, 268(E-Q)EGFP, 313(E-Q)EGFP, 323(D-N)EGFP and 326(D-N)EGFP are compared with OsNHX1-EGFP.

The sample of OsNHX1-EGFP (On the right, marked in red font) have problems.

Figure 4D

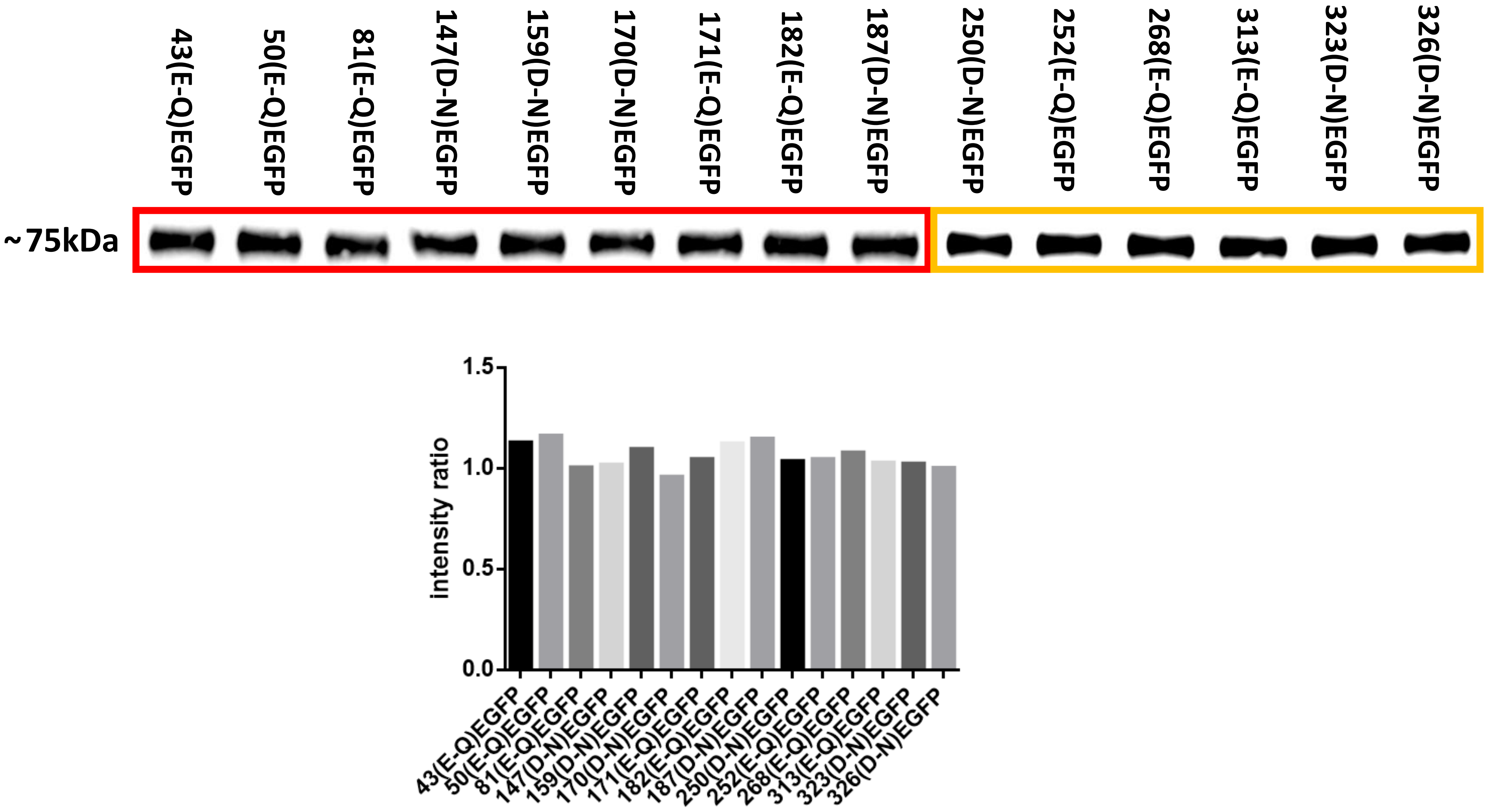

The intensity ratio of densitometry readings between the sample of 43(E-Q)EGFP, 50(E-Q)EGFP, 81(E-Q)EGFP, 147(D-N)EGFP, 159(D-N)EGFP, 170(D-N)EGFP, 171(E-Q)EGFP, 182(E-Q)EGFP, 187(D-N)EGFP, 250(D-N)EGFP, 252(E-Q)EGFP, 268(E-Q)EGFP, 268(E-Q)EGFP, 313(E-Q)EGFP, 323(D-N)EGFP, 326(D-N)EGFP and OsNHX1.

Original image

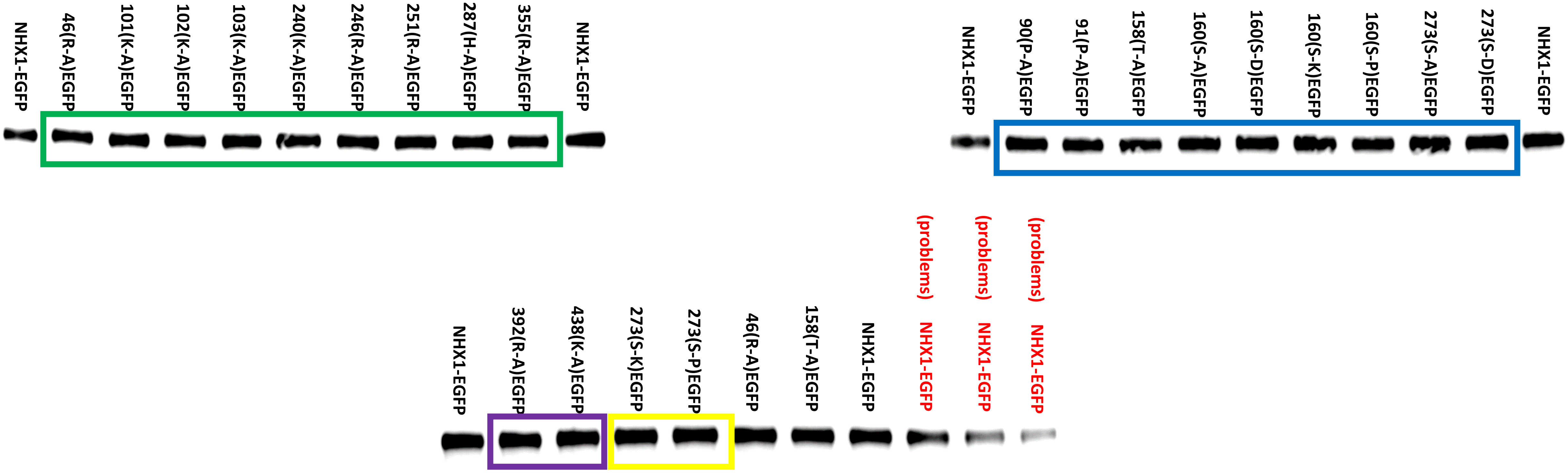

The microsomal membrane proteins (25 μg) were separated by SDS-PAGE, the SDS-PAGE gel (between 75kDa-100kDa) is cut, and was subjected to Western blot, using GFP antibody.

The sample of 46(R-A)EGFP, 101(K-A)EGFP, 102(K-A)EGFP, 103(K-A)EGFP, 240(K-A)EGFP, 246(R-A)EGFP, 251(R-A)EGFP, 287(H-A)EGFP, 355(R-A)EGFP, 392(R-A)EGFP and 438(K-A)EGFP are compared with OsNHX1-EGFP. The sample of 46(R-A)EGFP has been repeatedly confirmed.

The sample of 90(P-A)EGFP, 91(P-A)EGFP, 158(T-A)EGFP, 160(S-A)EGFP, 160(S-D)EGFP, 160(S-K)EGFP, 160(S-P)EGFP, 273(S-A)EGFP, 273(S-D)EGFP, 273(S-K)EGFP and 273(S-P)EGFP are compared with OsNHX1-EGFP. The sample of 158(T-A)EGFP has been repeatedly confirmed.

The sample of OsNHX1-EGFP (The rightmost section of the bottom image, marked in red font) have problems.

Figure 5D

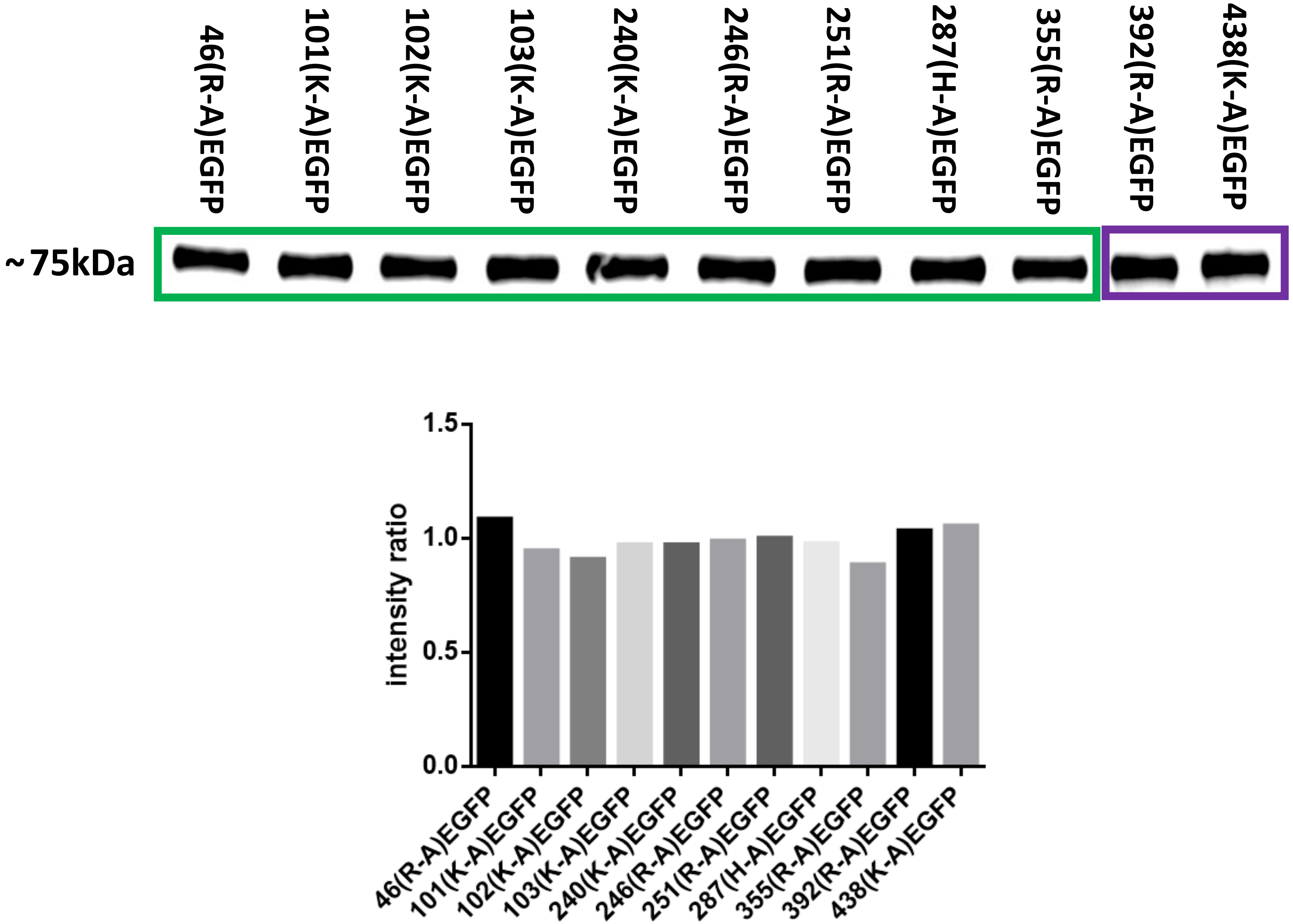

The intensity ratio of densitometry readings between the sample of 46(R-A)EGFP, 101(K-A)EGFP, 102(K-A)EGFP, 103(K-A)EGFP, 240(K-A)EGFP, 246(R-A)EGFP, 251(R-A)EGFP, 287(H-A)EGFP, 355(R-A)EGFP, 392(R-A)EGFP, 438(K-A)EGFP and OsNHX1.

Figure 6D

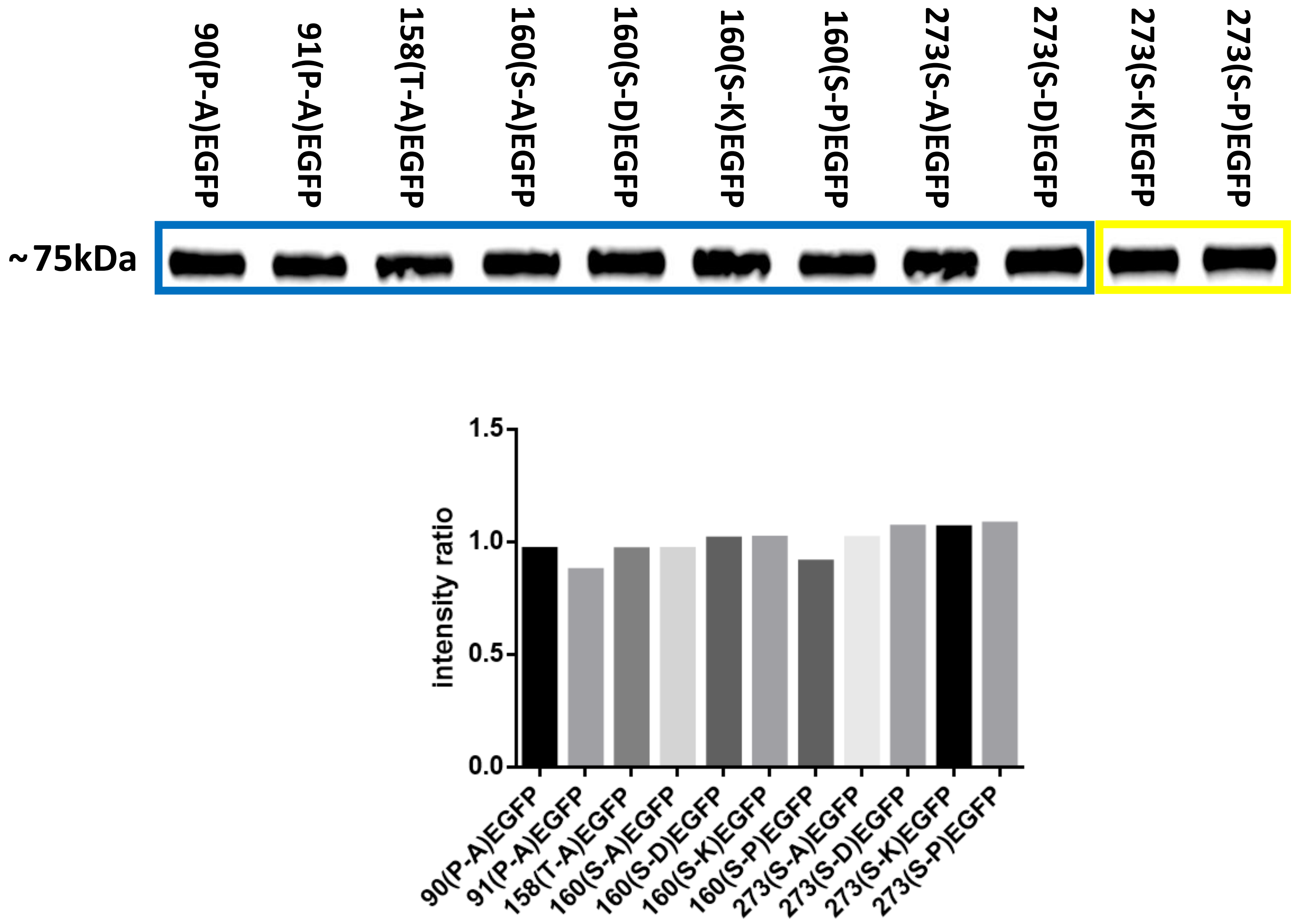

The intensity ratio of densitometry readings between the sample of 90(P-A)EGFP, 91(P-A)EGFP, 158(T-A)EGFP, 160(S-A)EGFP, 160(S-D)EGFP, 160(S-K)EGFP, 160(S-P)EGFP, 273(S-A)EGFP, 273(S-D)EGFP, 273(S-K)EGFP, 273(S-P)EGFP and OsNHX1.

Original image

Western blots figures with protein ladders

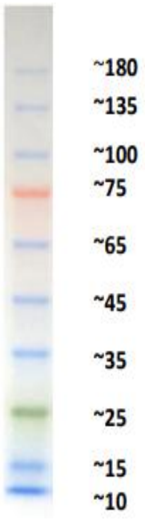

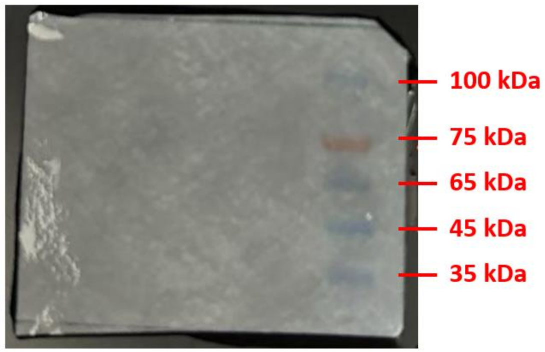

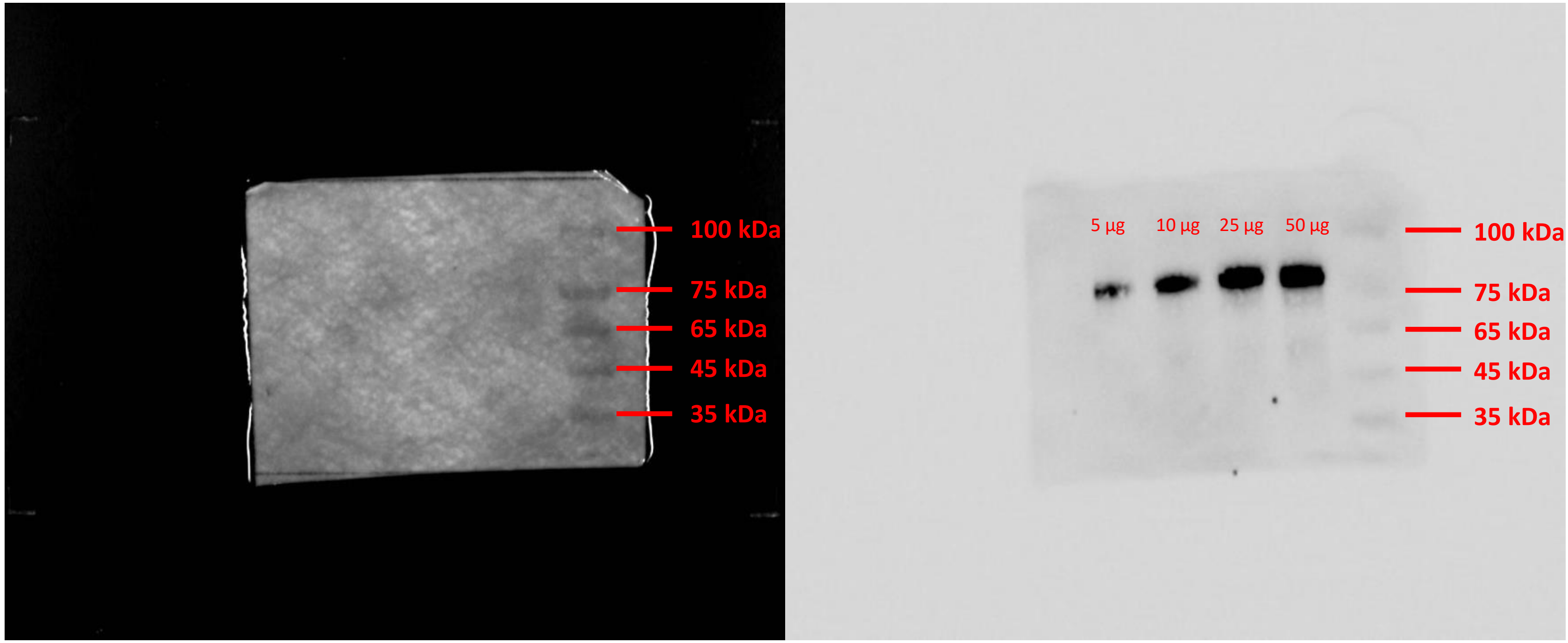

Figure 1

Figure 2

Figure 3

The western blots utilized the M5 prestained protein ladder from Mei5bio company (Catalog No. MF212) (Figure 1) and the GFP antibody from Beyotime company (Catalog No. AG281).

In preliminary experiments, to determine the appropriate loading amount, The microsomal membrane proteins of OsNHX1-EGFP (50 µg, 25 µg, 10 µg, and 5 µg) were separated by SDS-PAGE. After transfer, the M5 prestained protein ladder exhibited clearly defined protein size markers (Figure 2). Since the M5 prestained protein ladder does not exhibit intrinsic fluorescence, no significant fluorescence signal was observed after exposure (Figure 3). Nevertheless, the target protein band was approximately 75 kDa.
